# Accuracy, reliability, feasibility and nurse acceptance of a subcutaneous continuous glucose management system in critically ill patients: a prospective clinical trial

**DOI:** 10.1186/s13613-016-0167-z

**Published:** 2016-07-21

**Authors:** Tobias Wollersheim, Lilian Jo Engelhardt, Jeanne Pachulla, Rudolf Moergeli, Susanne Koch, Claudia Spies, Michael Hiesmayr, Steffen Weber-Carstens

**Affiliations:** Department of Anesthesiology and Operative Intensive Care Medicine, Campus Charité Mitte and Campus Virchow Klinikum, Charité – Universitätsmedizin Berlin, Augustenburger Platz 1, 13353 Berlin, Germany; Berlin Institute of Health (BIH), Berlin, Germany; Division Cardiac-, Thoracic-, Vascular Anesthesia and Intensive Care, Medical University Vienna, Vienna, Austria

**Keywords:** Continuous glucose monitoring, Subcutaneous, Interstitial, Critically ill patients, ICU, Medtronic Sentrino^®^, Accuracy, Reliability, Feasibility, Nurse acceptance, Evaluation

## Abstract

**Background:**

Continuous glucose monitoring (CGM) has not yet been implemented in the intensive care unit (ICU) setting. The purpose of this study was to evaluate reliability, feasibility, nurse acceptance and accuracy of the Medtronic Sentrino^®^ CGM system in critically ill patients.

**Methods:**

Sensors were inserted into the subcutaneous tissue of the patient’s thigh, quantifying interstitial glucose concentration for up to 72 h per sensor. Reliability and feasibility analysis included frequency of data display, data gaps and reasons for sensor removal. We surveyed nurse acceptance in a questionnaire. For the accuracy analysis, we compared sensor values to glucose values obtained via blood gas analysis. Potential benefits of CGM were investigated in intra-individual analyses of factors, such as glycemic variability or time in target range achieved with CGM compared to that achieved with intermittent glucose monitoring.

**Results:**

The device generated 68,655 real-time values from 31 sensors in 20 critically ill patients. 532 comparative blood glucose values were collected. Data were displayed during 32.5 h [16.0/62.4] per sensor, which is 45.1 % of the expected time of 72 h and 84.8 % of 37.9 h actual monitoring time. 21 out of 31 sensors were removed prematurely. 79.1 % of the nursing staff rated the device as not beneficial; the response rate was one-third. Mean absolute relative difference was 15.3 % (CI 13.5–17.0 %). Clarke error grid: 76.9 % zone A, 21.6 % zone B, 0.2 % zone C, 0.9 % zone D, 0.4 % zone E. Bland–Altman plot: mean bias +0.53 mg/dl, limits of agreement +64.6 and −63.5 mg/dl. Accuracy deteriorated during elevated glycemic variability and in the hyperglycemic range. There was no reduction in dysglycemic events during CGM compared to 72 h before and after CGM. If CGM was measuring accurately, it identified more hyperglycemic events when compared to intermittent measurements. This study was not designed to evaluate potential benefits of CGM on glucose control.

**Conclusions:**

The subcutaneous CGM system did not perform with satisfactory accuracy, feasibility, or nursing acceptance when evaluated in 20 medical-surgical ICU patients. Low point accuracy and prolonged data gaps significantly limited the potential clinical usefulness of the CGM trend data. Accurate continuous data display, with a MARD < 14 %, showed potential benefits in a subgroup of our patients.

*Trial registration* NCT02296372; Ethic vote Charité EA2/095/14

**Electronic supplementary material:**

The online version of this article (doi:10.1186/s13613-016-0167-z) contains supplementary material, which is available to authorized users.

## Background

Critically ill patients frequently experience stress-induced alterations in glucose homoeostasis resulting in hyperglycemia [1]. Peripheral insulin resistance and an enhanced hepatic glucose production, caused by a release of counter-regulatory hormones and cytokines, are contributing mechanisms [1, 2]. Insufficient GLUT4 translocation in skeletal muscle of critically ill patients is related to glucose dysregulation [3]. Hyperglycemia, elevated glycemic variability and hypoglycemia, were associated with an increased mortality risk in critically ill patients [4–6]. Randomized controlled trials showed that insulin therapy and management of glycemic control in the ICU remains challenging [6–10].

Continuous glucose monitoring (CGM) in the ICU, combined with an appropriate insulin protocol, may improve management of glycemic control and consequently impact patient outcome [11–13]. Wernerman et al. provided an overview of CGM technologies, including glucose oxidase, mid-infrared spectroscopy and fluorescence, ranging from invasive intravascular devices to minimally invasive interstitial and noninvasive transcutaneous systems [11]. Interstitial devices designed for use in diabetic patients have already been applied in critically ill patients [13–16]. Despite promising attempts, these systems have not yet been implemented to daily routine in the ICU and improvements are desirable. The subcutaneous Medtronic Sentrino^®^ CGM system was designed for use in ICU patients. The displayed real-time glucose trend line allows the ICU staff to observe glucose excursions at an earlier stage when compared to the established intermittent measurements. Patients may benefit from increased time in target range and improved glycemic variability. In addition, nurse workload may be reduced.

The purpose of this study was to evaluate reliability, feasibility, nurse acceptance and accuracy of this subcutaneous CGM system, as well as to identify potential weaknesses of the device in severely ill patients. In addition to previous studies [17–19], we retrospectively assessed potential benefits of CGM in comparison with intermittent glucose monitoring in our medical-surgical ICU.

## Methods

### Inclusion criteria and study participants

Inclusion criteria included an expected length of stay in the ICU of at least 72 h, age ≥18 years and written informed consent given by patient or legal proxy. We recruited critically ill patients during a time period of seven months in 2014. Patient inclusion started immediately after the local ethics committee, Ethikkommission Charité Universitätsmedizin Berlin, approved the study protocol (Charité-EA2/095/14). The protocol was registered under https://clinicaltrials.gov, trial registration number NCT02296372.

### Glycemic control in the study setting

The single-center study was set in two interdisciplinary mixed medical-surgical ICUs of a university hospital. The glucose target levels for insulin therapy were 80–149 mg/dl. Dysglycemic events were defined as follows: ranges above 149 mg/dl represented moderate hyperglycemia, and glucose levels above 179 mg/dl represented severe hyperglycemia. Moderate hypoglycemia was defined in a range from 41 to 70 mg/dl, and severe hypoglycemia as ≤40 mg/dl [11, 20]. Due to general ICU routine, nurses took blood samples from an arterial catheter in 2- to 4-h intervals, depending on the patient’s condition. In the absence of an arterial line, blood was collected from a central or peripheral venous catheter. Blood glucose was determined by glucose oxidase reaction using a Radiometer ABL 800 FLEX (Copenhagen, Denmark) blood gas analyzer. Depending on the identified blood glucose value, the nursing staff regulated the intravenous insulin therapy according to the local insulin protocol (Additional file 1: Table S1). All patient data, including blood gas analyses, were documented within the patient data management system (PDMS).

### CGM sensor

According to manufacturer’s information, the sensors of the interstitial CGM device consist of four independently working electrodes, which are embedded in two cannulas. This multisensory system provides enhanced signal stability and accuracy in critically ill patients. The electrodes are coated by glucose oxidase. In the enzymatic reaction, electrons are released and create an electrical gradient, which is proportional to the interstitial glucose concentration. Based on the electrical signal, the CGM algorithm calculates out of the four data signals one blood glucose value, which is displayed on a bed-sided monitor. The device provides one real-time glucose measurement per minute, with an insignificant lag time for signal processing, for up to 72 h (for more details, see Additional file 1: CGM Device).

### Study procedure

**Fig. 1:**
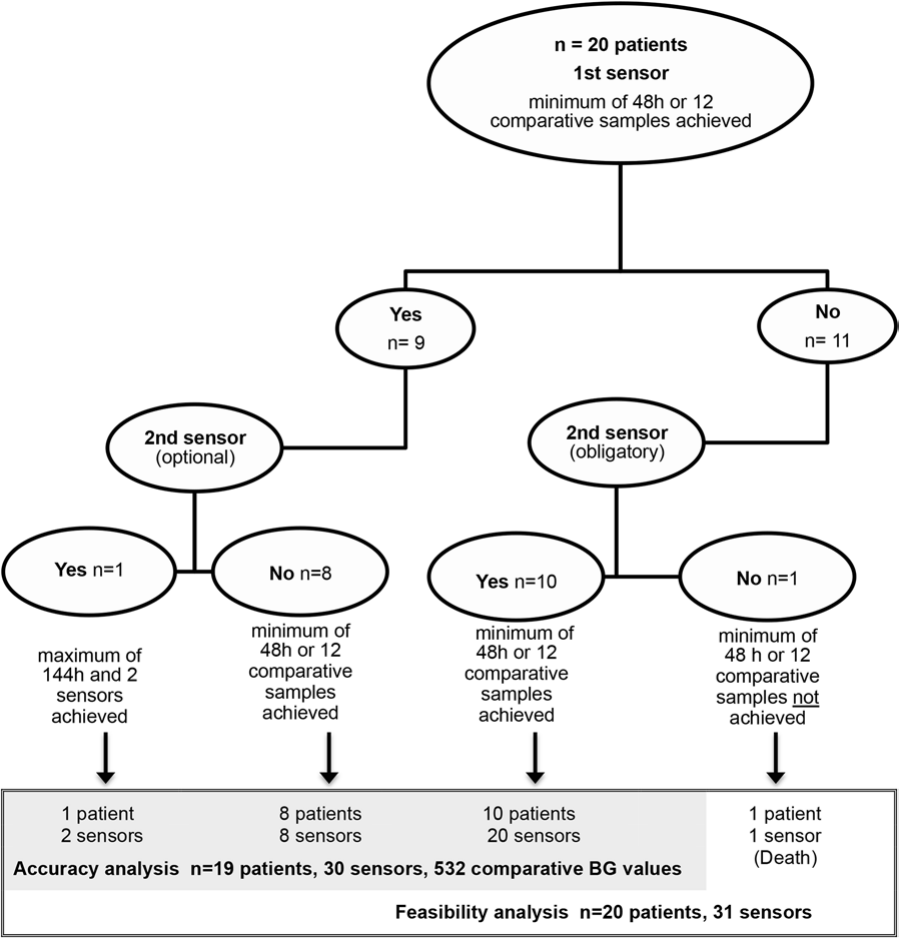
Study procedure. We included *n* = 20 patients during 57 days of recruiting. One patient was excluded from the accuracy analysis due to a lack of comparative blood glucose samples. Ten patients required a second sensor to achieve the minimum number of comparative samples or a minimum running time of 48 h. We used an optional second sensor in one patient

We inserted the sensors into the subcutaneous tissue of patient’s upper leg. After initialization, the sensors required one initial blood glucose entry, followed by two further calibrations after the first and second running hour. Subsequently, the study team performed calibrations every 8 h, as proposed by the manufacturer. The ward staff were not required to perform further calibrations. We instructed nurses to observe the continuous glucose trend line and perform blood glucose measurements to adapt insulin therapy in case of excursions above or below the target range (defined in the local insulin protocol Additional file 1: Table S1). Glucose values determined by the blood gas analyzer were used as reference. Blood glucose measurements used for initial calibrations and calibrations after data gaps (>15 min) were excluded from the point accuracy analysis. Further blood glucose measurements were included and compared to the latest CGM value immediately before calibration. As specified in the study protocol (Fig. 1), the accuracy analysis required a minimum monitoring time of 48 h or at least 12 comparative readings.

### Analysis criteria

**Fig. 2:**
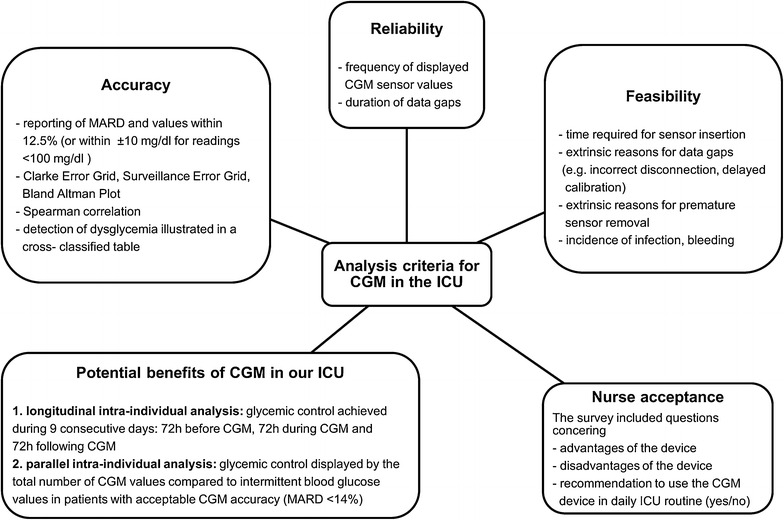
Analysis criteria. Detailed criteria for the evaluation of subcutaneous CGM in the ICU

Figure 2 illustrates detailed endpoints for the analysis of reliability, feasibility, nurse acceptance, accuracy and potential benefits of CGM. The analysis is based on the 2013 consensus recommendations, published by Finfer et al. defining criteria for continuous glucose control in critically ill patients [20]. Desirable reliability criteria include a continuous data display during >95 % of time and device-related data gaps <30 min [20]. We calculated frequency of data gaps and analyzed the gaps subdivided as very brief (<15 min), brief (15–30 min), prolonged (>30 min) and very prolonged (>2 h), so as to better describe the clinical significance of the missing trend data. The feasibility analysis considered the capacity of the device to perform within the busy ICU setting. This was supplemented by a survey of nurse acceptance assessed by brief questionnaires given to the nurses in charge of each shift (Additional file 1: Fig. S3). To determine accuracy, sensor values were compared to the simultaneously recorded blood glucose values from PDMS. We calculated point accuracy according to criteria specified within the consensus recommendations [20], which can be summarized as follows

- 98 % of device readings should be within 12.5 % of a reference standard (or within ±10 mg/dl for readings <100 mg/dl)
- The remaining 2 % of readings should be within 20 % of a reference standard
- Mean absolute relative difference (MARD) should be <14 % (M)ARD = │(blood glucose − sensor glucose)│/blood glucose × 100
- MARD > 18 % represents poor accuracy.

In addition, we analyzed possible confounding factors on MARD, such as arterial pO_2_, temperature, hemoglobin, potassium, lactate, pH value, sequential organ failure assessment (SOFA) Score, systemic inflammatory response syndrome (SIRS), history of diabetes, blood glucose variability and glucose ranges (<80 mg/dl, 80–179 mg/dl, >179 mg/dl). We retrospectively calculated MARD after time-shifting the reference a fixed amount (1 up to 30 min), so as to investigate a time delay as a possible confounding factor. To investigate potential benefits of CGM in our ICU, we report glycemic control achieved with CGM compared to that achieved with intermittent glucose monitoring. This was accomplished by performing intra-individual analyses, longitudinal and parallel, of factors such as mean blood glucose level, blood glucose variability, number of dysglycemia events and time in blood glucose target range (Fig. 2). Glycemic variability was determined using standard deviation of blood glucose and glycemic lability index (Table 4b), as a time-weighted index [21]. To evaluate safety, we reported local complications and discussed patient risks due to inaccurate CGM measurements in a safety statement.

### Statistical analysis

Results were shown as median with interquartile range or as absolute numbers with percentages. Clinical accuracy was illustrated using Bland–Altman plot [22], Clarke error grid [23] and Surveillance Error-Grid [24]. We calculated glycemic lability index using EasyGV© software [21]. Nonparametric tests were performed (Mann–Whitney *U* Test, Wilcoxon Test, Kruskal–Wallis Test, Friedman Test and Spearman’s correlation). We used IBM© SPSS© Statistics version 21, Microsoft Excel© ^2010^ and R for the statistical analysis.

## Results

We included 20 critically ill patients in this prospective trial using a total of 31 sensors (Fig. 1). Table 1 shows patient characteristics. In total, the device generated 68655 (1144.3 h) real-time glucose values during 1337.1 h of monitoring. The median monitoring time per patient was 70.5 h [57.2/72.7]. For the accuracy comparison, we collected 532 blood glucose values in 19 patients, of which 475 (89.3 %) were obtained from arterial and 57 (10.7 %) from venous catheters. There was no significant difference in accuracy between the 475 arterial blood glucose values compared to all 532 glucose values (*p* = .799). Table 2 shows a summary of glycemic control metrics.

**Table 1:** Patient characterization

Age	61 [54/69]
Gender (female/male)	14 (70 %)/6 (30 %)
BMI (kg/m^2)^	23 [22/26]
Diagnosis leading to ICU stay	
ARDS (ECMO, ECLA)	6 (30 %)
ARDS (without ECMO, ECLA)	8 (40 %)
Mediastinitis	1 (5 %)
Peritonitis	3 (15 %)
Intracranial hemorrhage	1 (5 %)
Polytrauma	1 (5 %)
At least one event of SIRS or sepsis during CGM	20 (100 %)
Sequential organ failure assessment (SOFA) Score at inclusion	8 [4/10]
Acute physiology and chronic health evaluation (APACHE) 2 Score at admission	24 [19/28]
History of diabetes mellitus	5 (25 %)
Administration of intravenous insulin therapy during CGM	14 (70 %)
Administration of vasopressors during CGM	7 (35 %)
Mean dose of epinephrine during sensor running time (µg/kg/min) (in seven patients receiving vasopressors)	0.08 [0.03/0.14]
Mortality during ICU stay	4 (20 %)

*n* = 20 patients

Results are expressed as median with interquartile range or as absolute numbers with percentages of *n* = 20 patients

*BMI* body mass index, *ARDS* acute respiratory distress syndrome, *ECMO* extracorporeal membrane oxygenation, *ECLA* extracorporeal lung assist, *SIRS* systemic inflammatory response syndrome, *SOFA* sequential organ failure assessment, *APACHE* acute physiology and chronic health evaluation, *ICU* intensive care unit, *CGM* continuous glucose monitoring

**Table 2:** Mean glucose level, glycemic variability and glycemic events

	Reference blood glucose	Comparative CGM reading	
Number of comparative glucose readings	532	532	
Readings per patient	28 [18/34]	28 [18/34]	
Mean glucose level per patient (mg/dl)	133.8 [128.4/147.5]	133.7 [124.3/150.1]*	
Glucose variability per patient measured in SD (mg/dl)	24.8 [19.9/35.2]	32.5 [25.2/42.2]^§^	
Glycemic events: number and percentage of *n* = 19 patients			
Severe hypoglycemia (≤40 mg/dl)	1 (5.3 %)	1 (5.3 %)	
Moderate hypoglycemia (41–70 mg/dl)	1 (5.3 %)	10 (52.6 %)	
Euglycemia (71–149 mg/dl)	19 (100 %)	19 (100 %)	
Moderate hyperglycemia (150–179 mg/dl)	18 (94.7 %)	19 (100 %)	
Severe hyperglycemia (>179 mg/dl)	15 (78.9 %)	11 (57.9 %)	
Glycemic events: number and percentage of *n* = 532 readings	158 (29.7 %)	188 (35.3 %)	
Severe hypoglycemia (≤40 mg/dl)	1 (0.2 %)	1 (0.2 %)	Chi-square test: *p* < 0.001 for crosstabulation see supplement
Moderate hypoglycemia (41–70 mg/dl)	2 (0.4 %)	15 (2.8 %)	
Euglycemia (71–149 mg/dl)	374 (70.3 %)	344 (64.7 %)	
Moderate hyperglycemia (150–179 mg/dl)	96 (18.0 %)	101 (19 %)	
Severe hyperglycemia (>179 mg/dl)	59 (11.1 %)	71 (13.3 %)	

*n* = 19 patients

Results are expressed as median with interquartile range or as absolute numbers with percentages

*SD* standard deviation

* *p* = 1.0; ^§^ *p* = 0.002

### Reliability, feasibility and safety

The reliability analysis showed a real-time data display during 32.5 h (16/62.4) per sensor, which is 45.1 % of the expected time of 72 h and 84.8 % of the 37.9 h actual monitoring time. During 80223 min (1337.1 h) of monitoring, we observed in total 11568 min (192.8 h) of missing values. The number of data gaps was 155, of which 68 (43.9 %) were very brief (<15 min), 35 (22.6 %) were brief (15–30 min), 27 (17.4 %) were prolonged (30–120 min) and 25 (16.1 %) were very prolonged. The sensor insertion itself was easily performed and required less than 10 min. The complication rate at the site was low. Minor bleeding after insertion occurred in four patients. We observed no local infection. The main feasibility issue was premature sensor removal. Detailed device reliability and feasibility is shown in Table 3.

**Table 3:** Reliability and feasibility

	Per sensor
Initialization time	27 min
Total time until first displayed value	37.5 min [36/42]
Expected monitoring time after initialization	72 h
Actual monitoring time after initialization	37.9 h [23/71.3]
Real-time data display	32.5 h [16/62.4]
Percentage of real-time data display/expected monitoring time after initialization	45.1 %
Percentage of real-time data display/actual monitoring time after initialization	84.8 % [67.9/93.8]
Data gaps after initialization	5 h [1.9/8.3]
Percentage of data gaps/actual monitoring time after initialization	15.2 % [6.2/32.1]
Number of performed calibrations	9.5 [6/13]

Reasons for the 11,568 min (192.8 h) of data gaps	Percentage of data gaps 11,568 min (192.8 h)	Percentage of monitoring time 80,223 min (1337.1 h)
1 Poor sensor signal (%)	23.3	3.4
2 Sensor failure (%)	15.0	2.2
3 Processor line error (%)	10.9	1.6
4 Disconnection (%)	15.6	2.3
5 Pending after reconnection (%)	3.4	0.5
6 Calibration required (%)	27.8	4.0
7 Others (%)	4.0	0.6
Device-related reasons (1–3) (%)	49.2	7.2
Not device related (4–7) (%)	50.8	7.4

Reasons for 21 prematurely sensor removals	Number of sensors	Percentage of all sensors (%)	Percentage of removed sensors (%)
Sensors 72 h completed	10	32.3	–
Sensors removed prematurely	21	67.7	100
1 Accidentally	7	22.6	33.3
2 Poor sensor signal during measurement	7	22.6	33.3
3 Poor sensor signal immediately after initialization	1	3.2	4.8
4 MRI	1	3.2	4.8
5 Discharge	1	3.2	4.8
6 Death	2	6.5	9.5
7 Others	2	6.5	9.5
Device-related reasons (1–3)	15	48.4	71.4
Not device related (4–7)	6	19.4	28.6

*n* = 20 patients, 31 sensors

Results are expressed as median with interquartile range or as absolute numbers with percentages

*MRI* magnetic resonance imaging

### Nurse acceptance

The nurses received 128 questionnaires during the CGM monitoring period. The response rate was one-third (*n* = 43, 34 %). The majority (79.1 %) of the nursing staff rated the device as not beneficial in the daily ICU routine. Advantages, such as the opportunity to observe glucose trends, were reported in 20.9 % of the questionnaires. Disadvantages were described by 53.5 %. Reasons included the inadequate alarm performance (23.3 %), the additional device (23.3 %) and device line (6.9 %) as disturbing factors during bedding and mobilization in the ICU routine.

### Point accuracy

**Fig. 3:**
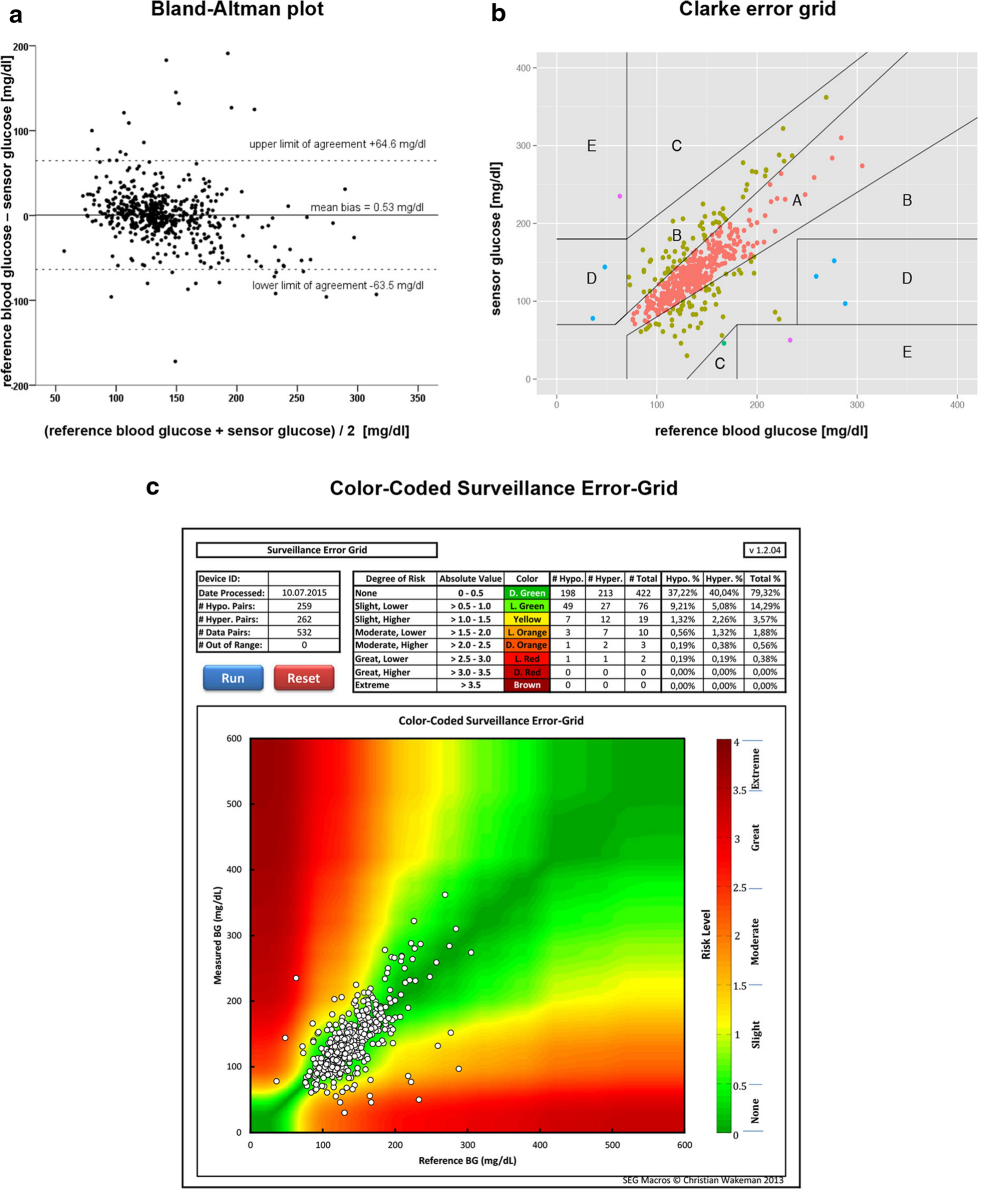
Bland–Altman plot, Clarke error grid, Color-coded Surveillance Error-Grid. *n* = 532 comparative samples. **a** Bland–Altman plot. The mean bias indicates whether there is a systematic error. Upper and lower limits were calculated by mean bias ±1.96 × standard deviation of the difference between BG and sensor glucose and represent random variations around the mean bias. If there is a Gaussian distribution, 95 % of points are located between these limits. [22, 41]. **b** Clarke error grid. Distribution: *A* = 76.9 %, *B* = 21.6 %, *C* = 0.2 %, *D* = 0.9 %, *E* = 0.4 %. Zones A (CGM data ≤20 % deviation from BG) and *B* are considered as clinically acceptable zones, whereas values in zones *C*, *D* and *E* are increasingly dangerous for the patient, and zone *E* may lead to adverse therapeutic decisions. [23]. **c** Color-coded Surveillance Error-Grid. The Surveillance Error-Grid software is available at http://www.diabetestechnology.org/SEGsoftware/Surveillance-Error-Grid-Analysis.xlsm. Last Accessed: Dec 11 2015 [24]

60.3 % of sensor data were within 12.5 % from the reference blood glucose (or were within ±10 mg/dl for readings <100 mg/dl). In total, 76.9 % of sensor readings were within 20, and 23.1 % deviated more than 20 % from the reference. MARD was 15.3 % (95 % CI 13.5–17.0 %). Spearman’s correlation coefficient was 0.688, *p* < .001, *r*^2^ = 0.461. The Bland–Altman plot (Fig. 3a) showed a mean bias of 0.53 mg/dl and limits of agreement of +64.6 mg/dl and −63.5 mg/dl. Clarke error grid and Color-Coded Surveillance Error-Grid (Fig. 3b, c) showed potentially dangerous errors. Additional file 1: Table S2 shows the detection of dysglycemic events.

### Confounding factors on accuracy

**Fig. 4:**
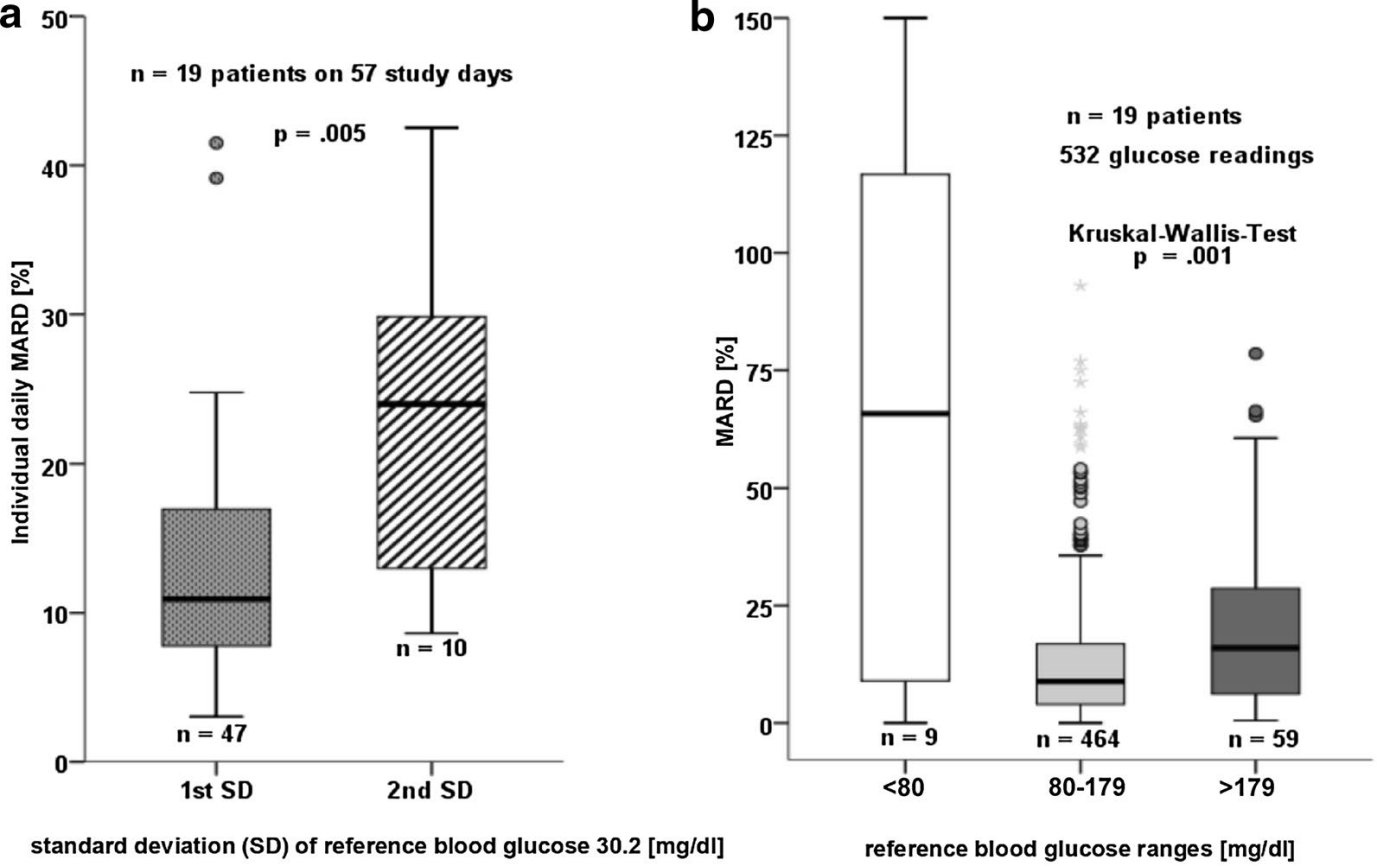
Confounding factors on MARD. **a** (*left*): Association between MARD and individual daily blood glucose variability shown in first and second standard deviation of reference glucose. *First boxplot* The CGM device shows acceptable accuracy* (MARD median 10.9 %) if the blood glucose variability is low (first standard deviation). *Second boxplot* Accuracy deteriorates (MARD median 24 %) during increased blood glucose variability (second standard deviation). **b** (*right*): Association between MARD and blood glucose ranges. *Second boxplot* The CGM device shows acceptable accuracy* (MARD median 8.8 %) in blood glucose ranges between 80 and 179 mg/dl. *First* and *Third boxplots* Accuracy deteriorates in the hypoglycemic range (MARD median 65.8 %) and during severe hyperglycemia (MARD median 16 %). *According to criteria specified within the consensus recommendations [20]*, MARD should be* <14 %

We identified that the blood glucose variability, analyzed in standard deviation, was significantly associated with CGM accuracy (Fig. 4a). Confirming this finding, standard deviation per patient was positively correlated with MARD per patient *k* = 0.593, *p* = .001, *n* = 19, *r*^2^ = 0.298 (Additional file 1: Fig. S1). MARD deteriorated in the hyperglycemic blood glucose range (Fig. 4a). There was no significant improvement or deterioration of MARD after time-shifting the reference glucose a fixed amount of 1 up to 30 min (Additional file 1: Fig. S2). MARD was worse during application of vasoconstrictors (Additional file 1: Table S3a). Previously known diabetes mellitus and episodes of SIRS did not confound MARD (Additional file 1: Table S3a). The severity of disease, measured via SOFA Score, showed a minor positive correlation with the MARD (*k* = 0.088, *p* = .043, *r*^2^ = 0.006, *n* = 532). There was no significant correlation of arterial pO2, temperature, pH value, lactate, hemoglobin, or potassium and MARD (Additional file 1: Table S3b).

### Potential benefits of CGM in our ICU

In 10 patients with an ICU stay of at least nine consecutive days, the longitudinal analysis showed no significant reduction in dysglycemic events during 3 days of CGM compared to 72 h before and 72 h after CGM (Table 4a). In the parallel analysis, CGM determined significantly lower minimal glucose values and detected more hyperglycemic events compared to intermittent blood glucose values in eight patients, in whom the device displayed accurate results with a MARD < 14 % (Table 4b).

**Table 4:** Potential benefits of CGM in our ICU

Per patient	Blood glucose 3 days before CGM	Blood glucose 3 days during CGM*	Blood glucose 3 days after CGM	*p* value
(a) Longitudinal analysis. Blood glucose metrics before/during/after CGM. *n* = 10 patients, 998 blood glucose values				
Number of BGA	30 [25/40]	35.5 [28/41]	26.5 [22/34]	*p* = .001^§^
Number of hypoglycemia	0 [0/0]	0 [0/0]	0 [0/0]	*p* = 1.00
Number of hyperglycemia	3 [1/4]	2.5 [1/5]	2 [1/3]	*p* = .779
Time in target (in %)	82.5 [74/98.2]	81.6 [68.9/95.6]	88.7 [81.3/94.3]	*p* = .452
Time <71 mg/dl (in %)	0 [0/0]	0 [0/0]	0 [0/0]	*p* = 1.00
Time >149 mg/dl (in %)	17.5 [1.8/26]	18.4 [4.4/31.1]	11.3 [5.7/15.4]	*p* = .717
Blood glucose min (mg/dl)	89 [80/100]	85 [73/106]	97.5 [85/110]	*p* = .273
Blood glucose max (mg/dl)	173.5 [162/187]	202 [159/218]	166 [153/185]	*p* = .014^#^
Mean glucose level (mg/dl)	134 [126.1/137.1]	130.7 [123.5/139]	128.5 [120.6/138.4]	*p* = .497
Mean glucose SD (mg/dl)	18.9 [15.8/22.4]	20.7 [17.6/36.4]	16.2 [11.6/24.2]	*p* = .741

Per patient	Blood glucose values (*n* = 239)	CGM sensor glucose (*n* = 34056)	*p* value
(b) Parallel analysis. Comparison of intermittent blood glucose to CGM glucose metrics including the total number of CGM readings, *n* = 8 patients MARD < 14 %, 239 blood glucose values, 32,044 CGM values			
Number of readings	29.5 [26.5/31.5]	3975 [3780/4109]	*p* = .012
Number of hypoglycemia	0 [0/0]	0.5 [0/2]	*p* = .066
Number of hyperglycemia	1 [1/5]	7 [6/18]	*p* = .018
Time in target range (in %)	88.7 [60.7/96.5]	85.2 [57.9/91.6]	*p* = .208
Time <71 mg/dl (in %)	0 [0/0]	0.3 [0/2.3]	*p* = .068
Time >149 mg/dl (in %)	11.3 [3.6/39.3]	14.3 [6.2/40.6]	*p* = .327
Glucose min (mg/dl)	103.5 [87/111.5]	76 [62/91]	*p* = .017
Glucose max (mg/dl)	195 [154.5/211]	186 [178.5/220.5]	*p* = .208
Mean glucose level (mg/dl)	130.2 [124.3/147.9]	128.7 [120.5/147.4]	*p* = .327
Mean glucose SD (mg/dl)	19.9 [14.4/22.7]	20.6 [16.5/28.4]	*p* = .093
Glycemic lability index	38.0 [14/53]	36.9 [18.5/90.7]	*p* = .674

Intra-individual longitudinal and parallel analysis, target range 71–149 mg/dl

Results are expressed as median with interquartile range or as absolute numbers with percentages

Glycemic lability index: time interval 1440 min = 24 h, glucose in mg/dl, sampling interval: blood glucose 120 min = 2 h, sensor glucose 1 min

Number of hypoglycemia or hyperglycemia: only events of newly developed hypoglycemia or hyperglycemia were considered in the analysis

*BGA* blood glucose analysis, *SD* standard deviation, *GLI* glycemic lability index

^§^Wilcoxon test before and during *p* = .123; before and after *p* = .05, during and after *p* = .005

^#^Wilcoxon test before and during *p* = .241, before and after *p* = .415, during and after *p* = .013

* BGAs during CGM data gaps and times of temporary system failure are included

## Discussion

### Reliability, feasibility and nurse acceptance

This prospective study was initially conducted with the intention of implementing a minimally invasive, simple to use CGM device in our ICU, in order to improve glycemic control. Unfortunately, application and performance were not as reliable as expected. Numerous sensors were removed prematurely, and the percentage of data gaps in relation to the expected sensor running time exceeded the time specified within the consensus recommendations of ICU experts [20]. The fact that we did not demand additional calibrations from the nursing staff and that they were not involved in troubleshooting may have contributed to the extent of data gaps and the poor performance. Since 7.4 % of data gaps were not device related, the data display during 85.6 % of the sensor running time after initialization could be corrected to 93 %. The high rate of accidentally removed sensors underlines the vulnerable use of the subcutaneous device in intensive care. This is supported by the opinion of our ICU nursing staff. More experience with a device may enhance feasibility. However, device-related issues, which are not improvable by experience, occurred frequently. Recently published investigations evaluating the same device reported minimal differences in reliability, but the clinically relevant results were concordant [17–19].

### Point accuracy and confounding factors

The subcutaneous device did not fulfill the suggested accuracy criteria for CGM in critically ill patients, specified within the consensus recommendations of ICU experts [20]. The distribution in the Clarke error grid [23] was unsatisfactory, as all 532 comparative readings of this analysis should have been located in zone A or B, preferably in zone A. The Surveillance Error-Grid [24], which promises to be closer to clinical routine, showed similar degrees of risk. In the Bland–Altman plot, the mean bias indicated that there was no systematic error [22]. However, 95 % of the values were within 128 mg/dl of the reference glucose. These wide limits of agreement illustrated a high random error [22]. The detection of dysglycemia was critical. The results considering MARD values within the 12.5 % range, and Clarke error grid and Bland–Altman plot are precisely consistent with those reported by Van Hooijdonk et al. [17]. Two further studies showed slightly better accuracy of the same system [18, 19]. Although specifically designed for ICU use, the investigated subcutaneous device failed to achieve comparatively accurate results in all recently published trials [17–19], as opposed to the CGM technologies quantifying glucose concentration in the vascular compartment of critically ill patients [25–28]. This leads to the conclusion that, with the intention to administrate insulin therapy, the subcutaneous glucose determination is not the proper method to estimate blood glucose levels during the acute phase of severe illness.

Inaccuracies may be attributed to a physiological time delay relating to the glucose diffusion from the plasma to the interstitial compartment [29, 30]. In healthy humans and diabetes patients, this time delay has been observed to range from 0 to 40 min in various studies summarized by Scuffi et al. [30]. Rebrin et al. found no evidence that physiological delays exceeded 5–10 min and argued that device-related processes are responsible for longer periods [31]. Moreover, Boyne et al. addressed the issue of random inter-sensor time discrepancies, which were quantitatively similar to physiological time delays, when comparing measurements of two subcutaneous sensors in the same individual [32]. Factors influencing the glucose diffusion rate, such as blood flow, peripheral microcirculation, and metabolic rate of subcutaneous tissue and adjacent cells, are all frequently altered in critically ill patients [11, 33]. We found no indication for a fixed time shift, but time delay and interstitial sensor accuracy may vary depending on the patient’s condition. There is evidence to support this hypothesis, since the use of vasopressors and a higher SOFA Score downgraded sensor accuracy in the present trial. In contrast, the accuracy of a subcutaneous CGM device was significantly improved in patients with septic shock compared to patients without sepsis [34]. Further studies cited that circulatory shock requiring norepinephrine therapy and impaired microcirculation had no influence on subcutaneous sensor accuracy [35, 36]. Variable subcutaneous oxygen concentration may interfere with the glucose oxidase. We did not investigate tissue paO2, but arterial paO2, as a correlating factor, had no clinically relevant impact on accuracy.

Glucose homeostasis is affected by the peripheral glucose uptake [33]. Inflammation may lead to an insufficient GLUT 4 translocation to sarcolemmal membrane [3]. This mechanism resulted in an impaired glucose supply in skeletal muscle cells in ICU patients [3]. A decreased glucose uptake was observed in adipocytes of septic rats [37]. We hypothesize that an insufficient GLUT 4 translocation may occur in subcutaneous tissue cells of critically ill patients. This may influence the accuracy of a subcutaneous CGM device, when compared to blood glucose. Consequently, it can still be assumed that subcutaneous CGM reflects actual insulin-dependent tissue glucose dynamics, which may be clinically relevant [32].

We identified that the sensor accuracy deteriorated in patients with elevated glycemic variability, as well as in the hyperglycemic range. Unfortunately, inaccuracies of CGM occurred particularly often when the need for CGM would have been most beneficial. Delayed diffusion processes become increasingly significant during rapid glucose oscillations [30, 33] and may contribute to the adverse influence of glucose variability and hyperglycemia on sensor performance. In healthy volunteers, the interstitial glucose was similar to venous glucose during steady-state conditions, but an increased time delay was observed when glucose levels were rapidly elevated by glucose infusion [38]. We could not confirm the findings reported by van Hooijdonk et al. that accuracy was influenced by a history of diabetes [17]. As already assumed in this study, inaccuracies in critically ill diabetic patients were possibly attributable to glucose fluctuations [17]. Although intravascular and interstitial space should be considered as different glucose compartments, the sensor technology requires blood glucose calibrations [33, 39]. This is a major concern, since sensor calibration during glucose alterations may subsequently cause and amplify inaccuracies [33].

### Safety statement

The local complication rate was acceptable, but critical safety issues arose as a consequence of inaccurate measurements. Clarke error grid and Surveillance Error-Grid showed potentially dangerous situations for the patients. Clinicians need to be aware of the fact that this device is not safe to guide insulin therapy. Even if used only to support common glucose control, this device can lead to confusing situations in the ICU routine of glucose management. As our experience showed, clinicians should always critically question the displayed CGM data.

### Potential benefits of CGM in our ICU

Glucose monitoring with the CGM system did not improve glycemic control in the longitudinal, intra-individual analysis. Low accuracy, as well as low nurse acceptance, may be potential reasons. Besides, the time in target of our severely ill patients with and without CGM was high. As a consequence, it may be difficult to demonstrate improved control even with a device that had reasonable accuracy. Conversely, if CGM was accurate, it showed potential benefits. In contrast to the findings of Brunner et al., glycemic variability was not significantly different when calculated from accurate continuous values as compared to less frequent blood glucose values [40]. If accurate CGM systems and adapted insulin protocols are implemented in the ICU, further research is required to evaluate long-term effects on clinical outcomes in RCTs. Insulin therapy guided by CGM did not impact on time in target range and glycemic variability in previous RCTs [13, 14, 40].

### Potential areas for improvements

- Calibration should only be performed during “steady-state” glucose levels, and not during rapid glucose fluctuations [33, 39] or adapted within a special calibration algorithm
- Improved fixation method or different localization to avoid accidental sensor removal
- Wireless device to avoid data gaps caused by occasional disconnection during bedding or mobilization, as well as accidental removals
- Integration of the continuous glucose display into the established patient monitor to reduce additional equipment
- Inclusion of a suggestion according to the local insulin treatment protocol into monitor

### Limitations

Firstly, this was a point accuracy analysis, in which only the concurrent blood glucose sample was considered. The reporting of glucose trending is not possible in this trial. Secondly, in the clinical setting we cannot exclude that there is a delay between the taking of a blood sample and the actual analysis via blood gas analyzer, where the time-point is documented [12, 20]. Thirdly, not all nurses were familiar with the device after the initial instructions provided by the manufacturer. Fourthly, the low response rate to the questionnaires may bias the results of the nurse acceptance survey. Fifthly, due to the low number of actual hypoglycemic events, there is a lack of evidence to draw a conclusion concerning the accuracy during hypoglycemia. It has to be stressed that this study was not designed to evaluate potential benefits of CGM on glucose control and there was no variation to the insulin protocol.

## Conclusion

The Medtronic System did not perform with satisfactory accuracy, feasibility or nursing acceptance when evaluated in 20 medical-surgical ICU patients. Low point accuracy and prolonged data gaps significantly limited the potential clinical usefulness of the CGM trend data. Future studies are required to determine the clinical value of the real-time Sentrino^®^ glucose trend data and alarms, using a validated nurse-driven insulin dosing algorithm in order to improve the safety and efficacy of blood glucose control in hospitalized patients.

## Additional file

10.1186/s13613-016-0167-z Supplementary Method. CGM Device. Supplementary Tables. **Table S1.** The local insulin protocol. **Table S2.** Detection of dysglycemic events. **Table S3a.** Confounding factors on MARD. **Table S3b.** Spearman´s correlation of paO2, temperature, lactate, pH-value, hemoglobin, potassium and SOFA-Score and MARD. Supplementary Figures. **Fig. S1.** Correlation of blood glucose variability per patient and MARD per patient. **Fig. S2.** MARD after time-shifting the reference a fixed amount (1 up to 30 minutes). **Fig. S3.** Nurse questionnaire.

## Abbreviations

ICU: intensive care unit
BG: blood glucose
CGM: continuous glucose monitoring
CI: confidence interval
ICU: intensive care unit
MARD: mean absolute relative difference
PDMS: patient data management system
RCT: randomized controlled trial
SOFA: sequential organ failure assessment
