# Supplementary material for: Accuracy, reliability, feasibility and nurse acceptance of a subcutaneous continuous glucose management system in critically ill patients: a prospective clinical trial
Source: Ann Intensive Care. 2016 Jul 21;6:70. doi: 10.1186/s13613-016-0167-z (PMC4954792; doi:10.1186/s13613-016-0167-z)
Supplement: Supplementary file 1 — 10.1186/s13613-016-0167-z Supplementary Method. CGM Device. Supplementary Tables. Table S1. The local insulin protocol. Table S2. Detection of dysglycemic events. Table S3a. Confounding factors on MARD. Table S3b. Spearman´s correlation of paO2, temperature, lactate, pH-value, hemoglobin, potassium and SOFA-Score and MARD. Supplementary Figures. Fig. S1. Correlation of blood glucose variability per patient and MARD per patient. Fig. S2. MARD after time-shifting the reference a fixed amount (1 up to 30 minutes). Fig. S3. Nurse questionnaire. [file 13613_2016_167_MOESM1_ESM.pdf]

Additional file 1

Supplementary Method

CGM Device

The interstitial CGM device includes a disposable sensor, a reusable processor line and a touchscreen monitor. According to manufacturer´s information, the sensors of the interstitial CGM device consist of four independently working electrodes, which are embedded in two cannulas. This multisensory system provides enhanced signal stability and accuracy in critically ill patients. The electrodes are coated by glucose oxidase. In the enzymatic reaction, electrons are released and create an electrical gradient, which is proportional to the interstitial glucose concentration. Based on the electrical signal, measured by the parallel working electrodes, the CGM algorithm calculates one "valid" glucose value, which is displayed on a bed sided monitor. In case of "poor sensor signal" the data display is temporary terminated. Reasons include a major bias between calibration glucose value and expected sensor glucose, sensor dislocation or a weak electrical signal. Calibrations may stabilize the signal. The monitor displays "sensor failure" if the alert "poor sensor signal" remains for four hours. Visual and audible alarms are generated in case of excursions of the trend line above or below the target range. The advanced Sentrino® CGM technology avoids drug interferences of about 100 frequently used drugs, including acetaminophen, in critically ill patients.

Supplementary Tables

Table S1. The local insulin protocol.

Procedure	Glucose level
Start intravenous insulin therapy	Moderate hyperglycemia > 149mg/dl
Give intravenous bolus of insulin	Severe hyperglycemia >179mg/dl
Stop intravenous insulin therapy	80mg/dl
Give intravenous bolus of glucose/dextrose	Moderate hypoglycemia <71mg/dl
Reduce nutrition	Moderate (>150mg/dl) or severe hyperglycemia (>180mg/dl) despite intravenous insulin infusion of max. 6-8IU/h
**Perform arterial blood gas analysis to control blood glucose every two to four hours and 30minutes after changes in insulin infusion.**	

Table S2. Detection of dysglycemic events. n=532, 19 patients

Reference blood glucose [mg/dl]	CGM glucose readings [mg/dl]					
	**Glucose Range**	**Severe Hypoglycemia ≤40mg/dl**	**Moderate Hypoglycemia 41-70mg/dl**	**Euglycemia 71-149mg/dl**	**Moderate Hyperglycemia 150-179mg/dl**	**Severe Hyperglycemia ≥180mg/dl**
	**≤ 40mg/dl**	**0**	**0**	**1**	**0**	**0**
	**41-70mg/dl**	**0**	**0**	**1**	**0**	**1**
	**71-149mg/dl**	**1**	**12**	**311**	**38**	**12**
	**150-179mg/dl**	**0**	**2**	**24**	**48**	**22**
	**≥180mg/dl**	**0**	**1**	**7**	**15**	**36**

Out of 188 displayed dysglycemic events, 104 (55.5%) were incorrect, including one hyperglycemia during actual hypoglycemia. The device missed 3/3 (100%) hypoglycemic events, and failed to simultaneously display 71/155 (45.8%) hyperglycemic events. The Chi-Square Test showed a significant difference in distribution between the detected dysglycemic events by reference method and CGM readings.

Chi-Square Tests

	Value	df	Asymp. Sig. (2-sided)
Pearson Chi-Square	273.827	16	.000
Likelihood Ratio	244.912	16	.000
Linear-by-Linear Association	197.132	1	.000
N of Valid Cases	532		

Table S3a. Confounding factors on MARD. n=532 values, 19 patients

	Number of readings	MARD	p value (t test)
SIRS	453	15.7% (95% CI 13.7-17.8)	p=.137
No SIRS	79	12.5% (95% CI 9.3-15.7)	
Vasopressors	191	18% (95% CI 14-22)	p=.001*
No Vasopressors	431	13.7% (95% CI 12.1-15.3)	
Diabetes mellitus	112	15.8% (95% CI 12.8-18.7)	p=.888
No diabetes mellitus	420	15.1% (95% CI 13-17.2)	

Results are expressed as mean with 95% confidence interval (CI).

Abbreviations: Mean absolute relative difference (MARD), Systemic inflammatory response syndrome (SIRS)

Table S3b. **Spearman´s correlation of paO2, temperature, lactate, pH-value, hemoglobin, potassium and SOFA-Score and MARD**. n=532 values, 19 patients.

	**MARD**	**paO2**	**temperature**	**lactate**	**pH-value**	**Hemoglobin**	**Potassium**	**SOFA Score**
**MARD Spearman-Rho k**	1	-.089	-.049	.064	-.051	.081	-.023	.088*
**p-value**		.054	.266	.139	.245	.063	.589	.043

Venous blood gas analyzes were excluded from the correlation of paO2 and MARD

Abbreviations: Mean absolute relative difference (MARD), Sequential Organ Failure Assessment (SOFA) Score

Supplementary Figures

Fig. S1. Correlation of blood glucose variability per patient and MARD per patient

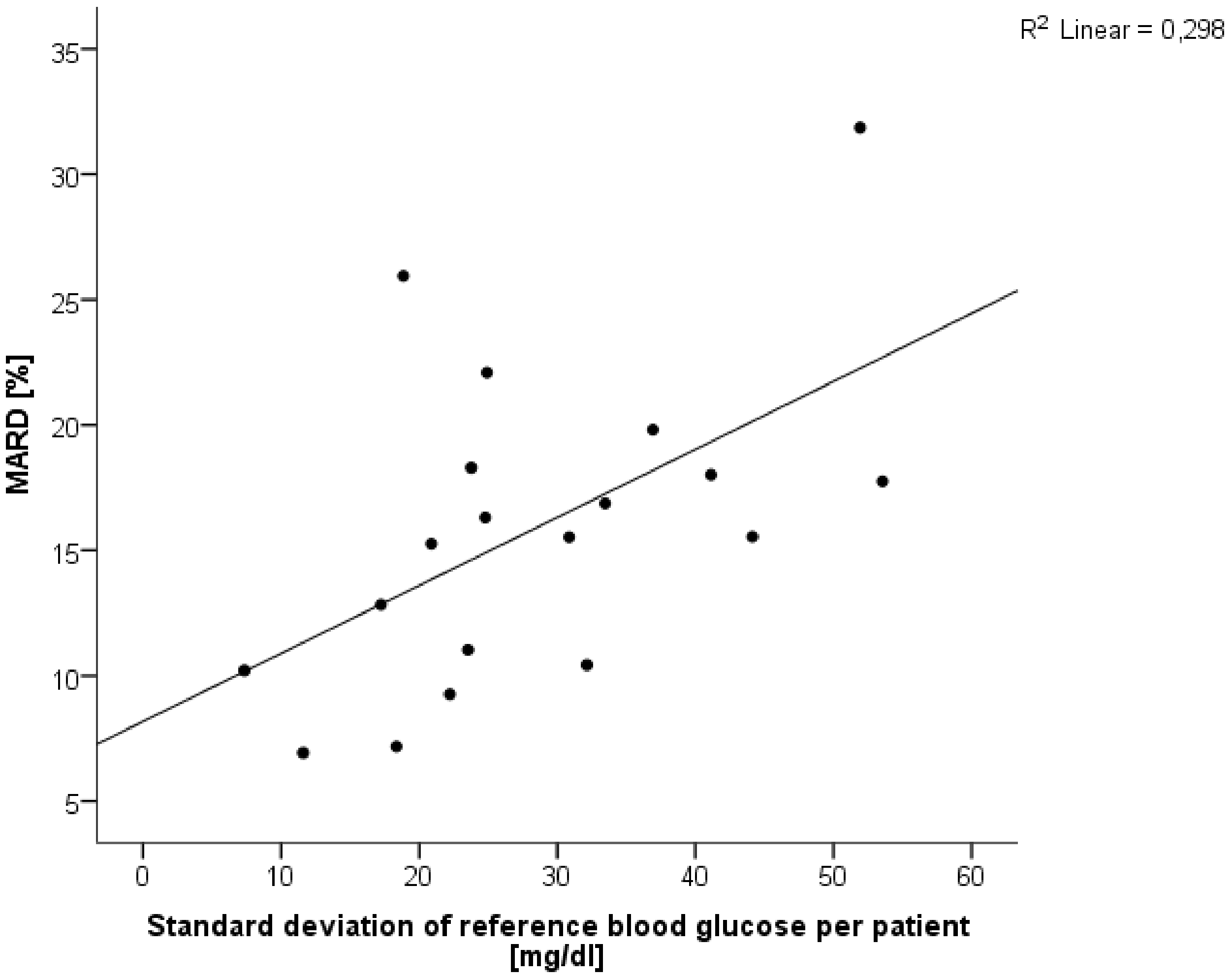

Glycemic variability measured in standard deviation of blood glucose. n=19, k=.593, p=.001, r^2=0.298.

Fig. S2. MARD after time-shifting the reference a fixed amount (1 up to 30 minutes)

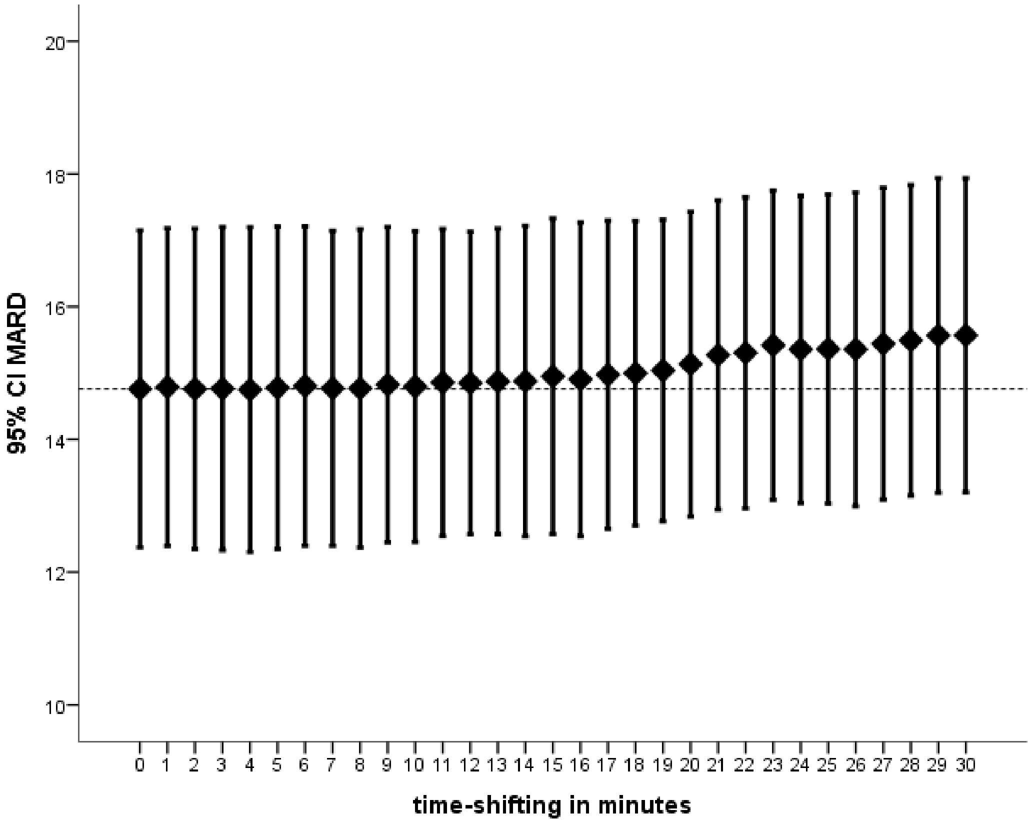

n=19 patients, 305 reference glucose values, 9455 CGM values. There was no significant improvement or deterioration of MARD after time shifting the reference glucose a fixed amount of 1 up to 30 minutes. Even after 30 minutes, MARD was not significantly different (p=.107) compared to time point 0.

Fig. S3. Nurse questionnaire

Nurse acceptance of MedtronicSentrino® CGM in the daily ICU setting - Questionnaire for the nursing staff

Date: ________________ ☐ Early shift ☐ Late shift ☐ Night shift

Do not use the device for therapeutic decisions. Perform changes in insulin therapy only after controlling blood glucose levels with the blood gas analyzer.

**Medtronic Sentrino® CGM**
**Is the use of MedtronicSentrino® CGM in this shift beneficial?**
☐ yes ☐ no if yes, name advantages? ....
**Is the use of MedtronicSentrino® CGM in this shift disadvantageous?**
☐ yes ☐ no if yes, name disadvantages? ....
**In case of accidental sensor removal, what was the reason?**
☐ Sensor removed by the patient<br>☐ Nursing care (bedding, washing)<br>☐ Mobilization<br>☐ surgery, CT, MRI<br>☐ Others: ....
**Would you recommend to use Medtronic Sentrino® CGM in the ICU in the future?** ☐ yes ☐ no
